# Frequency selection rule for high definition and high frame rate Lissajous scanning

**DOI:** 10.1038/s41598-017-13634-3

**Published:** 2017-10-26

**Authors:** Kyungmin Hwang, Yeong-Hyeon Seo, Jinhyo Ahn, Pilhan Kim, Ki-Hun Jeong

**Affiliations:** 1 Department of bio and brain engineering, KAIST, Daejeon, 34141 Republic of Korea; 2 Graduate School of Nanoscience and Technology, KAIST, Daejeon, 34141 Republic of Korea; 3 KAIST Institute for Health science and technology, Daejeon, 34141 Republic of Korea

## Abstract

Lissajous microscanners are very attractive in compact laser scanning applications such as endomicroscopy or pro-projection display owing to high mechanical stability and low operating voltages. The scanning frequency serves as a critical factor for determining the scanning imaging quality. Here we report the selection rule of scanning frequencies that can realize high definition and high frame-rate (HDHF) full-repeated Lissajous scanning imaging. The fill factor (FF) monotonically increases with the total lobe number of a Lissajous curve, i.e., the sum of scanning frequencies divided by the great common divisor (GCD) of bi-axial scanning frequencies. The frames per second (FPS), called the pattern repeated rate or the frame rate, linearly increases with GCD. HDHF Lissajous scanning is achieved at the bi-axial scanning frequencies, where the GCD has the maximum value among various sets of the scanning frequencies satisfying the total lobe number for a target FF. Based on this selection rule, the experimental results clearly demonstrate that conventional Lissajous scanners substantially increase both FF and FPS by slightly modulating the scanning frequencies at near the resonance within the resonance bandwidth of a Lissajous scanner. This selection rule provides a new guideline for HDHF Lissajous scanning in compact laser scanning systems.

## Introduction

Controlled steering of laser beams attracts assorted optical applications such as advanced optical microscopy^1,2^, 3D material processing^3,4^ including 3D printing^5,6^, single-pixel camera^7^, or screen-less display^8–10^. Recently, compact laser scanners become actively engaged in miniaturized imaging^11–15^ or pico-projection display^16,17^ systems. Laser microscanners such as microelectromechanical systems (MEMS) mirror scanners^18^ and resonant fiber piezoelectric tube (PZT) scanners^14,19^ are actively utilized for compact optical applications. MEMS mirrors have high design flexibility for the scanning speed as well as the batch fabrication but they still have some intrinsic limitations in realizing compact packaging schemes for forward-viewing applications. In contrast, the resonant fiber scanners mounted inside a quadrupole PZT allow the facile compact packaging. Recently, the microscanners move to clinical *in-vivo* endomicroscopic imaging for optical biopsy, where laser scanning of high definition and high frame rate within persistence of human vision is imperative in achieving clinically acceptable image resolution and real-time imaging^20,21^. High definition allows diffraction-limited endomicroscopic imaging of high resolution for visualizing morphological characteristics of tumor cells^22^. In addition, high frame rate apparently reduces motion artifacts^13^ as well as laser exposure dose on a tissue sample for less photo-bleaching and photo damages of live cells during *in-vivo* fluorescence imaging^23^. However, attaining both the requirements are still challenging, particularly for laser microscanners.

Laser scanning patterns such as spiral, raster, and Lissajous scans substantially affect both the image resolution and the frame rate of scanned images. A spiral scan is obtained by using two driving signals of the same frequency and 90° phase-shift on the orthogonal axes under the amplitude modulation along the radial direction. The frame rate of spiral scanning is simply determined by the amplitude modulation frequency. However, the non-uniform illumination densities over the field-of-view (FOV) occur as the scanning speed increase along the scan radius^12^. Besides, high illumination density at the center region readily causes photo-damage or photo-bleaching^23^. A spiral scan is often achieved by using a resonant bare-fiber scanner^24^. A raster scan provides a rectangular FOV by using two different driving signals of fast and slow scanning frequencies in integer multiple relationship. The frame rate is subject to the slow scanning frequency, which should be increased up to the persistence of human vision for real-time imaging. A raster scan is often achieved by using torsional silicon micromirrors with a gimbal mount or fiber scanners. However, torsional micromirrors require highly flexible micro-springs with a low resonant frequency for the slow motion^25^ and fiber scanners require a relatively large operating voltage for the slow motion to achieve enough field-of-view in static mode^26,27^. Such microscanners with high operating voltages are inappropriate for clinical applications^28^. Moreover, some microscanners with electrostatic actuation become more susceptible and have a large footprint size due to a large number of comb teeth^29^. As a result, the microscanners with raster scanning are intrinsically vulnerable to an external shock and they have some technical limitations for compact packaging. In contrast, a Lissajous scan is realized by using two arbitrary fast scanning frequencies along the bi-axial axes in the resonant mode and thus Lissajous microscanners have both high mechanical stability and low operating voltages^21^.

The scanned image quality of Lissajous scanning can be evaluated by several parameters such as the fill factor (FF), i.e., a ratio of scanned area over a total pixel area in a single image, the scanning density, the step size, and the frame rate, also called the frames per second (FPS). The scanned image mainly depends on bi-directional scanning frequencies, which are mainly equal to the resonant frequencies of a Lissajous microscanner. In particular, the FF and the FPS serve as the most critical factors because they are directly determined by the scanning frequencies. The FF increases when the ratio of scanning frequencies becomes more complex rational number, but a pattern repeated rate becomes very low, thus Lissajous scans are often utilized for slow laser scanning applications far below the temporal sensitivity of human vision^30–33^. Alternatively, non-repeating Lissajous scanning, partially scanned over the entire FOV, can offer the frame-rate faster than the full repeating rate of a Lissajous curve^34^. However, the non-repeating Lissajous scanning exacerbates the critical trade-off between the fill factor and the frame rate^35^. Moreover, conventional methods of the frequency selection reply on the non-repeating Lissajous scanning, which is applicable for some limited conditions that the phase difference between the x-axis and the y-axis should be π/2^36^. Moreover, temporal flickering becomes inevitable in obtaining real-time imaging because the Lissajous scan varies at every repeating cycle. Alternatively, some additional efforts on image reconstruction algorithms such as triangle based interpolation^37^ or model-based image reconstruction^35^ realize high frame rate but they also still have some technical imitations in achieving real-time imaging of complex patterns such as small pulmonary vessels and bronchi^38–40^.

**Figure 1:**
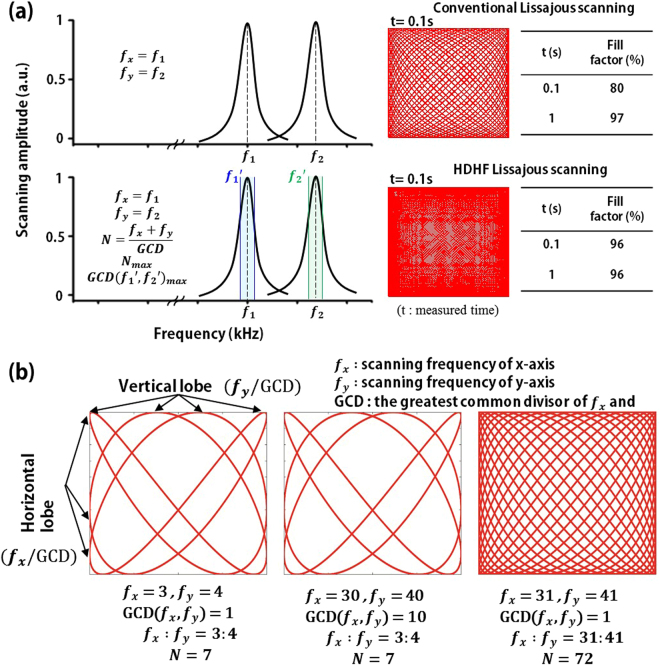
Comparison between conventional and high definition high frame rate (HDHF) Lissajous scanning methods. (**a**) Examples of the selection of scanning frequencies and the corresponding scanning patterns. Unlike conventional Lissajous scanning selecting the scanning frequencies at the resonance of a Lissajous scanner, HDHF Lissajous scanning chooses the scanning frequencies at near the resonant frequencies but within the resonance bandwidth, which can substantially increase the fill factor (FF) as well as the frame rate (FPS). For instance, a Lissajous pattern in conventional Lissajous scanning shows the fill factor of 80% for 0.1 sec at 1319 Hz and 1410 Hz in scanning frequency. In contrast, the scanning pattern in HDHF Lissajous scanning increases up to the fill factor of 96% for 0.1 sec by slightly modulating the scanning frequencies, i.e., 1310 Hz and 1410 Hz, which are still at near the resonance but within the resonant bandwidth. (**b**) Lissajous scanning patterns depending on the scanning frequencies. The greatest common divisor (GCD) of bi-axial scanning frequencies (f_x_ and f_y_) determines the FPS and also the total lobe number, i.e., the sum of horizontal and vertical lobe number, decides the fill factor. A particular set of scanning frequencies allows substantial increase of both the FPS and the FF for HDHF Lissajous scanning.

Here we report the selection rule of scanning frequencies for high definition and high frame-rate (HDHF) full-repeated Lissajous scanner. Figure 1a compares conventional and HDHF Lissajous scanning methods. Conventional Lissajous scanning often selects the scanning frequencies at the resonance of a Lissajous scanner. In contrast, HDHF Lissajous scanning selects the scanning frequencies at near the resonant frequencies within the resonance bandwidth, which can substantially increase the fill factor (FF) as well as the frame rate (FPS). For instance, a Lissajous pattern in conventional Lissajous scanning has a non-repeating cycle, which shows the FF of 80% for a scanning time of 0.1 sec at the scanning frequencies of 1319 Hz and 1410 Hz. However, HDHF Lissajous scanning can increase the FF up to 96% by using slightly different scanning frequencies of 1310 Hz and 1410 Hz at near the resonance and has a same scanning path at every frame. Figure 1b illustrates Lissajous scanning patterns depending on the scanning frequencies. The greatest common divisor (GCD) of bi-axial scanning frequencies (f_x_ and f_y_) directly determines FPS and also the total lobe number, i.e., the sum of horizontal and vertical lobe number, decides FF. A particular set of scanning frequencies results in substantial increase of both FPS and the FF that allows HDHF Lissajous scanning.

**Figure 2:**
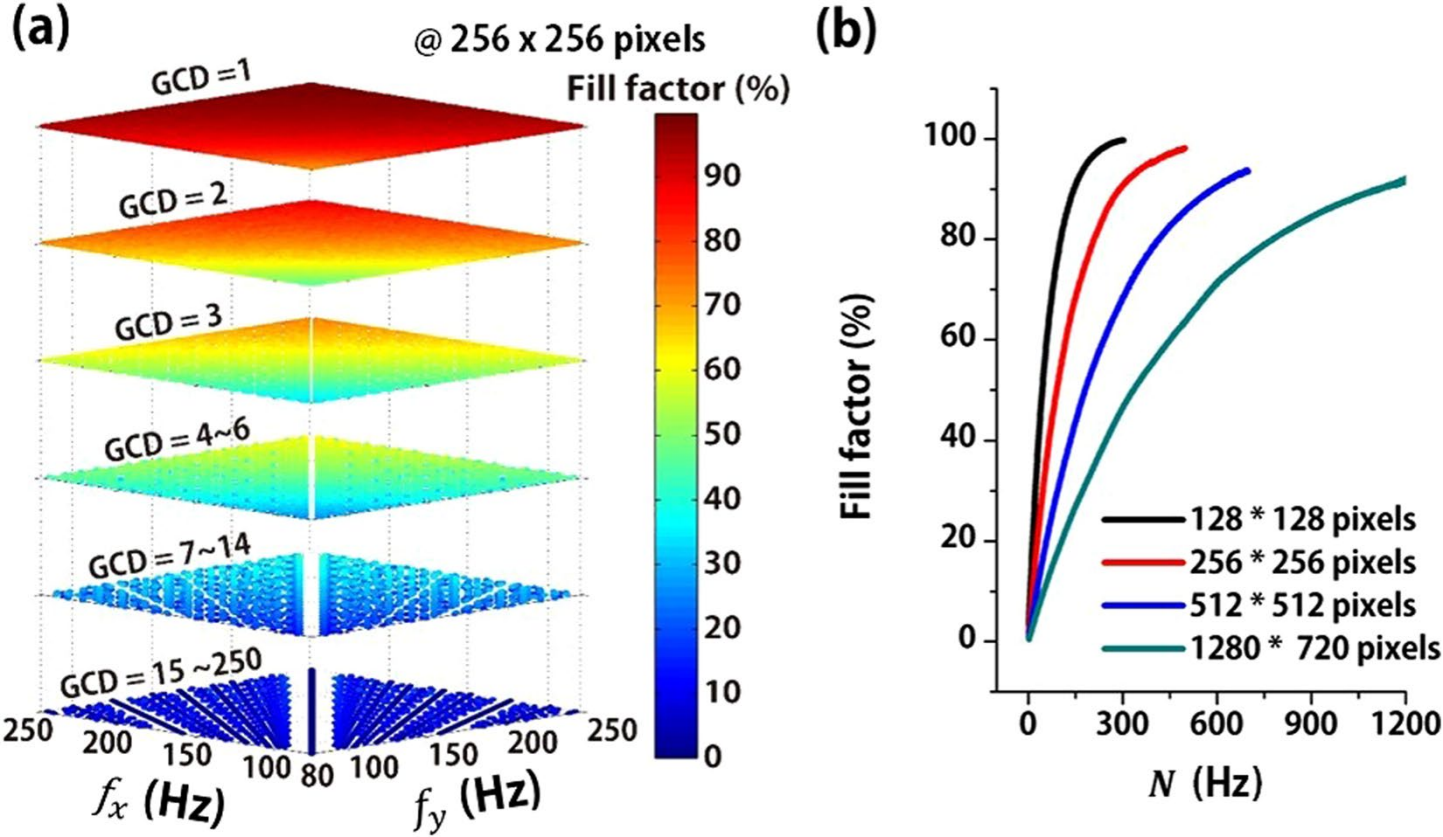
Frequency selection rule for HDHF Lissajous scanning. (**a**) The FF for a single shot image of 256 × 256 pixels depending on different scanning frequencies from 80 to 250 Hz. For bi-axial scanning frequencies with GCD = 1, the FF increases with the scanning frequency. However, low FF sporadically appears at scanning frequencies with high GCD. (**b**) The FF depending on total lobe number, i.e., N = (f_x_ + f_y_)/GCD, for 128 × 128, 256 × 256, 512 × 512, and 1280 × 720 pixels. The FF has a positively monotonic relationship with the N. In other words, the FF increases with the total lobe number but becomes saturated as the Lissajous curve fills the FOV within the limited number of image pixels.

A Lissajous curve can be calculated using x(t) = sin (2π f_x_ · t + φ_x_) and y(t) = sin (2π f_y_ · t + φ_y_), where f_x_ and f_y_ are the scanning frequencies, t is the scanning time, and φ is the corresponding phase for each axis. x(t) and y(t) indicate the position of a scanning beam over a scanning time. The FF was calculated using the number of image pixels passed through the Lissajous curve for a single frame. Figure 2a displays a scatter plot of the FF for a single shot image of 256 × 256 pixels depending on different scanning frequencies from 80 to 250 Hz. The scatter plots of the FF for 128 × 128, 512 × 512, and 1280 × 720 image pixels are shown in Fig. S1 (see the Supplementary information). For bi-axial scanning frequencies of co-prime, i.e., GCD = 1, the FF increases with the scanning frequency. However, scanning frequencies with high GCD sporadically produce the scanning patterns with a low FF. As shown in Fig. 2b, the calculated result shows the FF for different image pixels has a positively monotonic relationship with the total lobe number, i.e. N = (f_x_ + f_y_)/GCD. In other words, the FF increases with the total lobe number but becomes saturated as the Lissajous curve fills the FOV within the limited number of image pixels. Table 1 indicates the minimum total lobe number N_MIN_ (FF) that satisfies a specific FF for various image pixels. More detail tables about N for different target fill factors are also shown in Table S1 (see the Supplementary information). The FPS is also given by GCD when the both scanning frequencies are integers. As a result, HDHF Lissajous scanning can be achieved at scanning frequencies, where the GCD has the maximum value among various sets of scanning frequencies satisfying the target FF. In other words, the selection rule can be expressed by N ≥ N_MIN_ (FF) and Max [GCD (f_x_, f_y_)], where FF is the target fill factor and N is (f_x_ + f_y_)/GCD.

**Table 1:** The minimum total lobe number (N_MIN_) for various image pixels depending on the fill factor.

FF(%)	128 × 128	256 × 256	512 × 512	1280 × 720
70	78	154	320	581
75	90	177	365	668
80	103	204	416	778
85	120	237	488	911
90	146	284	594	1082
95	186	358	769	1346

**Figure 3:**
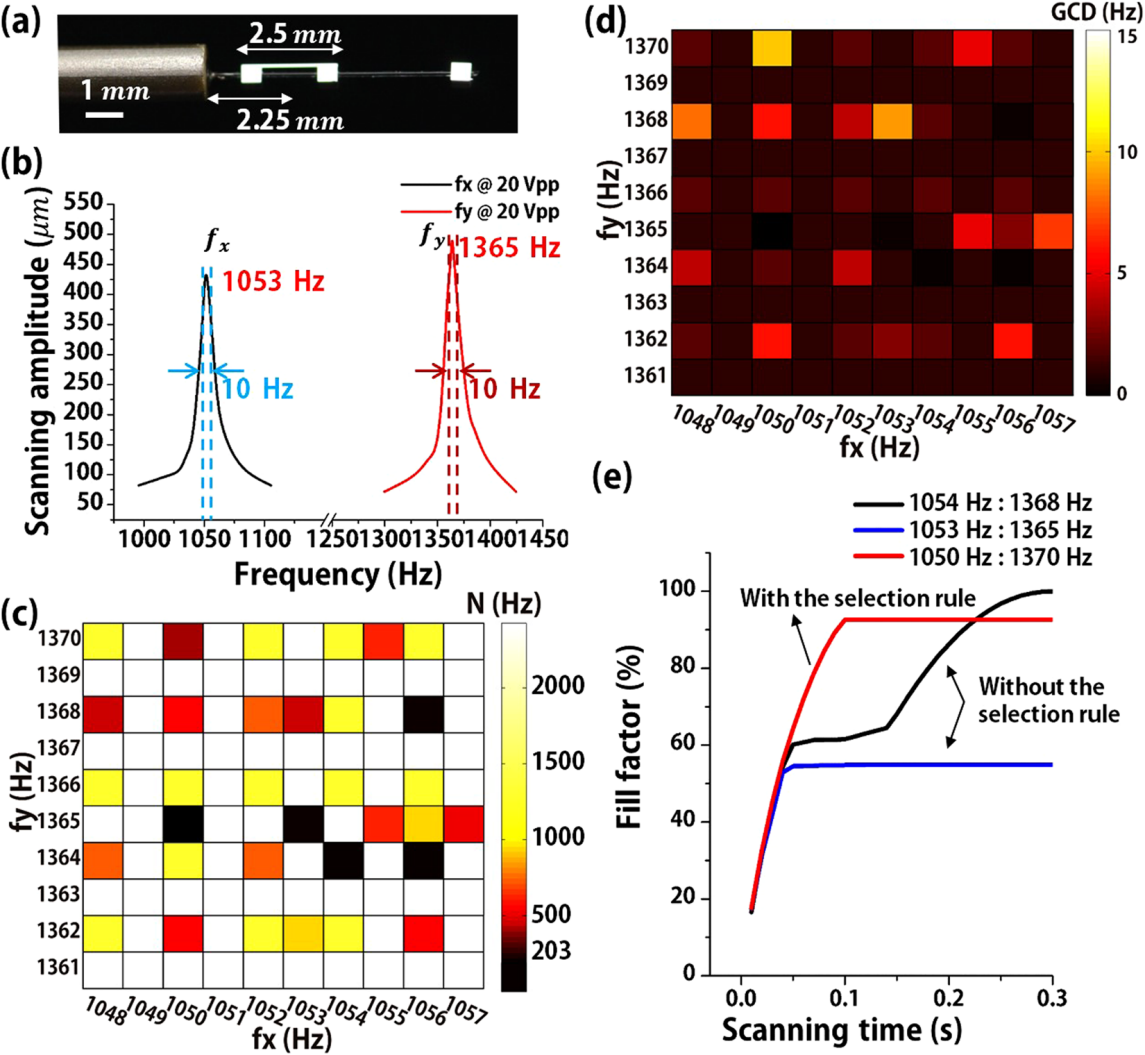
Experimental Demonstrations of HDHF Lissajous scanning using a Lissajous fiber scanner. (**a**) Assembled PZT based Lissajous fiber scanner. A silicon mass was precisely placed at the end of a single optical fiber. The location and length of the microspring were set to 2.25 mm and 2.5 mm, respectively. (**b**) Frequency response of the fiber scanner. The resonant frequencies are 1,053 Hz and 1,365 Hz for each axis, respectively, whose the resonant bandwidth is 10 Hz, respectively. (**c**) A color-map of total lobe number depending on the scanning frequencies, where the maximum (N ≥ 2,000) and the minimum (N ≤ 203) indicate yellow to black in color, respectively. (**d**) A color map of the GCD depending on the scanning frequencies. The specific areas of N < N_MIN_ = 204 are filled in black color. (**e**) Calculated results of the FF for microscanners with and without the selection rule depending on a scanning time. Lissajous scanning substantially increases both FF and FPS by slightly modulated within the bandwidth resonant frequencies of ±5 Hz. The scanning frequencies for HDHF Lissajous were finally determined as 1,050 Hz and 1,370 Hz for N = 242 and GCD = 10.

The frequency selection rule was experimentally verified by using a Lissajous fiber scanner. The FPS was set to 10 Hz with over 80% in FF for 256 × 256 image pixels because conventional Lissajous microscanners have 2~3 Hz in FPS^19,31,41^. Based on Table 1, the minimum total lobe number is 204 and the target FPS of 10 Hz is equal to the GCD. Based on the above selection rule, N = (f_x_ + f_y_)/10 ≥ N_MIN_ (80) = 204. Consequently, the sum of bi-directional frequencies turns out to be over 2,040 Hz. A stable Lissajous fiber scanner of ~ 1 kHz resonant frequencies for x-and y-axes was prepared with the micromachined tethered silicon oscillator (MTSO)^19^, which was fabricated by using deep-reactive-ion-etching (DRIE) on a silicon-on-insulator (SOI) wafer (see Supplementary, Fig. S2). The Lissajous fiber scanner consists of a lead zirconate titanate (PZT) tube, an optical fiber, and the MTSO. The MTSO consists of a microspring and a silicon mass of 0.5 mm × 0.5 mm × 0.4 mm. The position and length of microspring were set to 2.25 mm and 2.5 mm in order to differentiate the bi-directional resonant frequencies, respectively (Fig. 3a). The silicon mass was precisely placed at the distal end of 7 mm long optical fiber to increase FOV. Figure 3b shows the frequency response of a fully assembled Lissajous fiber scanner. The resonant frequencies are 1,053 Hz and 1,365 Hz for each axis, respectively. Note that the bi-axial scanning frequencies were slightly modulated within the bandwidth resonant frequencies of ±5 Hz, which still exceed 80% of the maximum scanning amplitude. Figure 3c displays a color-map of total lobe number depending on the scanning frequencies within the frequency range, where the maximum (N ≥ 2,000) and the minimum (N ≤ 203) indicate yellow to black in color, respectively. Figure 3d also describes a color map of the GCD depending on the scanning frequencies. The specific areas of N < N_MIN_ = 204 are filled in black color to satisfy the above equation. The scanning frequencies for HDHF Lissajous scanning were finally determined as 1,050 Hz and 1,370 Hz for N = 242 and GCD = 10. In addition, the FF and the FPS were also calculated by using two sets of scanning frequencies not satisfying the frequency selection rule (Fig. 3e). At the resonant operation of 1,053 Hz and 1,365 Hz, the FPS becomes high (GCD = 39) but the FF becomes very low because the N is only 62. The scanning frequencies of 1,054 Hz and 1,368 Hz obtain high FF due to high N but the FPS shows only 2 Hz.

**Figure 4:**
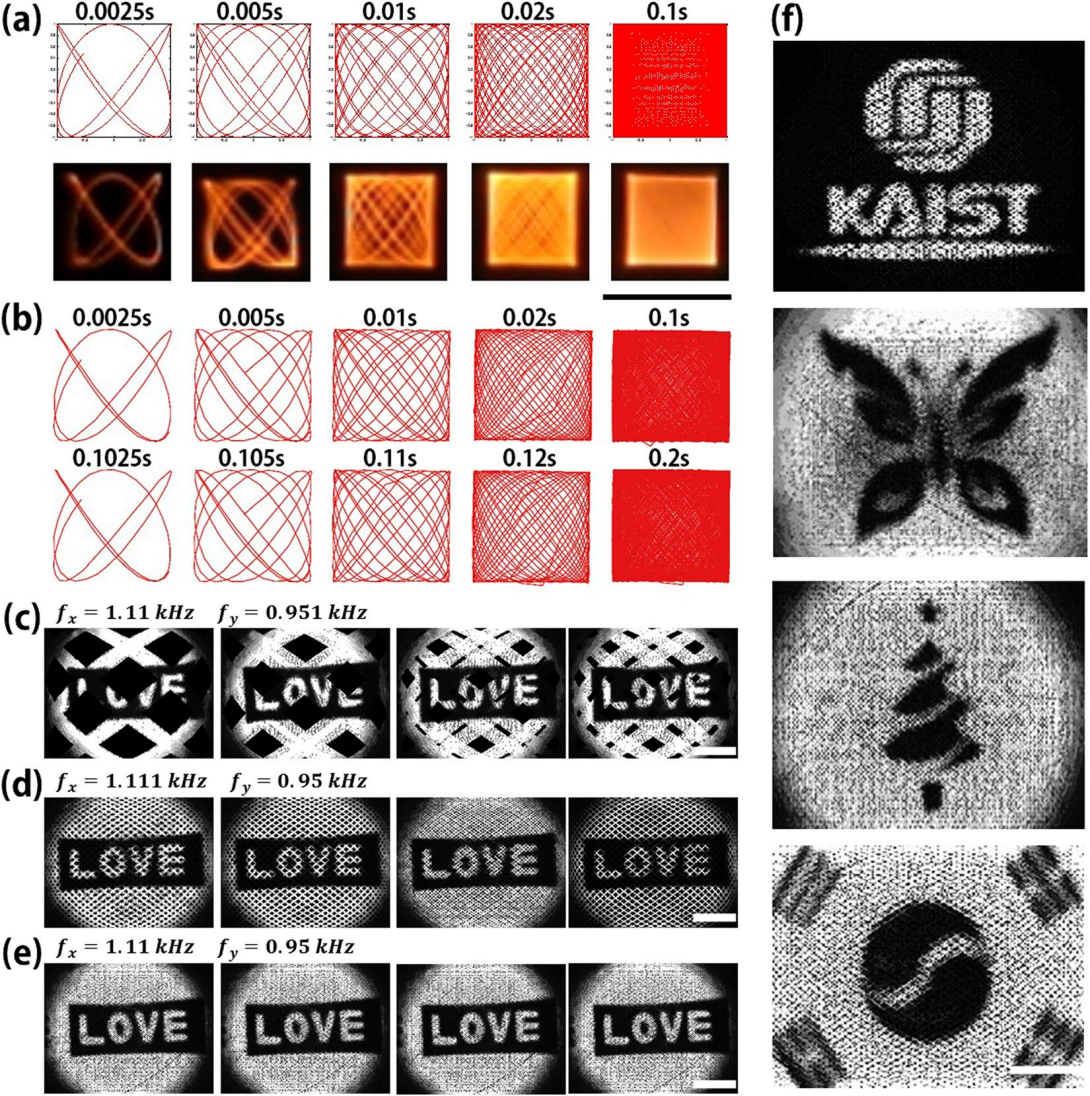
HDHR Lissajous scanning demonstration. (**a**) Ideal Lissajous patterns and optical Lissajous curves captured at 2.5, 5, 10, 20, 100 msec in order. (Scale bar = 400 um). Scanning frequencies are 1,050 Hz and 1,370 Hz at 20 V_pp_. (**b**) Beam tracking graphs from 0 second to 0.1 second and from 0.1 second to 0.2 second using ultrafast speed camera. Fill factors are 4.5**%**, 9**%**, 17**%**, 31.8**%**, and 92**%**, respectively. (**c**–**e**) 2D reflectance images captured at every 100 msec from real-time imaging of metal pattern “LOVE” at 256 by 256 pixels, obtained with 10 Hz in FPS using a fabricated Lissajous fiber scanner. Resonant frequencies of the fiber scanner are 1109 Hz and 951 Hz for x- and y-axes, whose the resonant bandwidth is 10 Hz, respectively. (**c**) Reflection images obtained at scanning frequencies of 1.11 kHz and 0.951 kHz with of 687 lobes and 3 Hz in FPS, respectively (Video 1). (**d**) Reflection images obtained at scanning frequencies of 1.111 kHz and 0.95 kHz with total number of 2,061 lobes and 1 Hz in FPS (Video 2). (**e**) Reflectance images obtained at scanning frequencies of 1.11 kHz and 0.95 kHz has no temporal flickering and shows high FF and high FPS (Video 3). (Scale bar = 50 μm) (**f**) 2D reflectance images from metal micropatterns using a Lissajous fiber scanner based on the HDHF Lissajous selection rule at 10 Hz in FPS. (Scale bar = 50 μm).

The HDHF Lissajous scanned imaging has been successfully demonstrated using Lissajous fiber scanners. The beam trajectory for the HDHF scanning was obtained at scanning frequencies of 1,050 Hz and 1,370 Hz. Time-lapse images for a single frame image were captured for 2.5~100 msec. Figure 4a depicts the calculated Lissajous images and the captured optical images for 2.5, 5, 10, 20, to 100 msec, respectively. Figure 4b indicates beam-tracking graphs obtained by using a high-speed camera (Fastcam Mini UX100, photron) from 0 sec to 0.1 sec and 0.1 sec to 0.2 sec, respectively. Both the calculated and experimental results clearly demonstrate that the Lissajous patterns exhibit similar scanning trajectories. The experimental results also demonstrate the beam tracking patterns for 0 msec to 2.5 msec have the same patterns with those for 100 msec to 102.5 msec and thus the Lissajous scanning pattern repeats every 0.1 sec. Besides, the fill factors are 4.5%, 9%, 17%, 31.8%, and 92% for each time in order. The HDHF selection rule was also applied for a different fabricated fiber scanner to obtain confocal reflection images. Figure 4(c–e) show 2D reflectance images captured at every 100 msec from micro-patterned metal pattern “LOVE” with 256 by 256 pixels using a home-built confocal reflectance imaging system (see the Supplementary, Fig. S4). The resonant frequencies of a Lissajous fiber scanner are 1.111 kHz and 0.951 kHz for x- and y-axes, respectively. The Lissajous fiber scanner obtained three different 2D reflectance images for the same target using three scanning frequencies which differ from the resonance frequency by only 1 Hz because the variation of 1 Hz in scanning frequency significantly affects both the total lobe number and the FPS. Figure 4c also demonstrates some reflection images obtained at scanning frequencies of 1.11 kHz and 0.951 kHz with of 687 lobes and 3 Hz in FPS, i.e. GCD (1110,951) = 3, respectively (Video 1). Based on Table 1, the Lissajous scan has the FF over 95% every 1/3 seconds, which is still slow for real-time imaging applications. When restoring an image with a frame-rate faster than 3 Hz, the pattern changes every frame as shown in the Fig. 4c resulting in temporal flickering. Figure 4d shows the reflection images obtained at scanning frequencies of 1.111 kHz and 0.95 kHz with total number of 2,061 lobes and 1 Hz in FPS (Video 2). This Lissajous scanning is still unsuitable for real-time imaging because the scanning patterns varies every frame. However, Fig. 4e, showing the reflectance images at scanning frequencies of 1.11 kHz and 0.95 kHz, exhibits high FF and high FPS without temporal flickering (Video 3). The scanning frequencies for Fig. 4e were in accordance with the frequency selection rule (see the Supplementary, Fig. S3) to satisfy over 80% in FF and 10 Hz in FPS. Figure 4f shows 2D reflectance images of various metal patterned samples using the HDHF Lissajous fiber scanner.

In summary, this work reports the general scanning frequency selection rule for HDHF full-repeated Lissajous scanners. HDHF Lissajous scanning can be achieved by selecting the bi-axial scanning frequencies with the highest FPS among various sets of scanning frequencies, which should be located at near the resonant frequencies within the resonance bandwidth and also satisfy the minimum N for a target FF. Based on this selection rule, the experiment results successfully demonstrate a fiber scanner exhibits HDHF Lissajous scanning, i.e., 10 Hz in FPS and 92% in FF at near the scanning frequency of 1 kHz, and furthermore 2D reflectance confocal imaging without any temporal flickering. This selection rule can provide a new guideline for obtaining HDHF Lissajous scanning in real-time and high-resolution compact laser scanning applications.

## Methods

### Fill factor depending on scanning frequencies

The fill factor depending on scanning frequencies was calculated using x(t) = sin(2πf_x_·t + φ_x_) and y(t) = sin(2πf_y_·t + φ_y_), where f_x_ and f_y_ are the scanning frequencies, t is the scanning time, and φ_x_ and φ_y_ are the corresponding phases. x(t) and y(t) give the location information about Lissajous scanning trajectory depending on the scanning time. The location information was coordinated for the trajectory position using the equations of X(t) = A_x_·[(x(t) + 1)/2] and Y(t) = A_y_·[(y(t) + 1)/2], where X(t) and Y(t) are x-coordinate and y-coordinate for the trajectory, and A_x_ and A_y_ are the number of pixels in vertical direction line and pixels in horizontal direction line, respectively. The X(t) and Y(t) were rounded into integer coordinates of Lissajous trajectory depending on the scanning time. Not only the scanning frequencies but also the phases affect to the trajectory of Lissajous, but the phases does not affect to resonant operation of micro-scanners and also can be adjust simply by a function generator. The fill factor was calculated using the number of pixels passed by the rounded X(t) and Y(t). Final fill factor is an average of fill factor results with 200 random phases and same scanning frequencies. The scanning frequencies were converted into the total lobe number (N), which is the sum of the two frequencies of bi-directional axes divided by the GCD. Consequently, the fill factor becomes the function of the N.

### Lissajous fiber scanner fabrication

HDHF Lissajous scanning has been experimentally demonstrated by using a Lissajous fiber scanner consisting of a lead zirconate titante (PZT) tube, an optical fiber, a silicon mass, and a micromachined tethered silicon oscillator (MTSO). Based on the above selection rule, the sum of bi-directional frequencies turns out to be over 2,040 Hz. A stable Lissajous fiber scanner requires similar spring constants for x- and y- axes of the fiber scanner. As the spring constant determines the resonant frequencies, ~1 kHz resonant frequencies for x-and y-axes of the Lissajous scanner was prepared with the MTSO, which was micromachined by using deep-reactive-ion-etching (DRIE) on 6-inch silicon-on-insulator (SOI) wafer. First, the bottom side of a silicon-on-insulator wafer was passivated with photoresist. Second, the MTSO were photolithographically defined with photoresist and etched by a DRIE process. The top side of the SOI wafer was then passivated with a thick photoresist. Third, a silicon groove for micro-assembly with a single optical fiber and the MTSO was further etched by using DRIE on the bottom silicon layer. After oxygen plasma ashing of the passivation photoresist layer, the silicon structures were released by removing the buried oxide layer in vapored hydrogen fluoride. Individual MTSOs were finally separated from the SOI wafer by breaking off the silicon tethers with Joule heating. The silicon mass structure of MTSO has the physical dimension of 0.5 mm × 0.5 mm × 0.4 mm and the silicon spring is 50 μm in width.

### Confocal imaging system setup

The laser (LDM series 785, LASOS) was fiber based laser for convenient fiber coupling to the laser. The maximum output power of the laser is 60 mW and FC/APC fiber connector was combined. Dichroic beam splitter (FF01-Di02, Semrock) reflected the guide laser and transmitted the 785 nm laser for same optical paths of both lasers. For detector, photomultiplier (PMT: H9305-03, Hamamatsu) and socket (R9110, Hamamatsu) were used. To control the gain of the PZT, controller (C4900-01, Hamamatsu) was used. The Lissajous fiber-scanning probe was combined to the system using two fiber collimators (PAFA-X-4B, Thorlabs). Each fiber collimator combined the laser and the scanning probe to the confocal system. (See the Fig. S4 in the Supplementary information).

## Electronic supplementary material

Video 1

Video 2

Video 3

Supplementary information
