# Supplementary material for: Frequency selection rule for high definition and high frame rate Lissajous scanning
Source: Sci Rep. 2017 Oct 26;7:14075. doi: 10.1038/s41598-017-13634-3 (PMC5658369; doi:10.1038/s41598-017-13634-3)
Supplement: Supplementary file 4 — Supplementary information [file 41598_2017_13634_MOESM4_ESM.pdf]

Supplementary information

Frequency selection rule for high definition and high frame rate Lissajous scanning

Kyung-Min Hwang[1, 3], *Yeong-Hyeon Seo*[1, 3], *Jinhyo Ahn*[2, 3], *Pilhan Kim*[2, 3], *and Ki-Hun Jeong*[1, 3,*]

[1]Department of bio and brain engineering, KAIST, Daejeon, 34141, Republic of Korea
[2]Graduate School of Nanoscience and Technology, KAIST, Daejeon, 34141, Republic of Korea
[3]KAIST Institute for Health science and technology, Daejeon, 34141, Republic of Korea
[*]kjeong@kaist.ac.kr

1. Fill factor depending on scanning frequencies

Figure S1 shows scatter plots of the fill factor for 128 x 128, 512 x 512 and 1280 x 720 image pixels depending on scanning frequencies. Based on the data of Fig. S1, the fill factors for various image pixels with 51% to 100% are analyzed for the total lobes number (N) values and expressed in the table S1.

The scanning frequencies of HDHF Lissajous scanning are selected as the frequencies with the largest GCD among the various sets of scanning frequencies satisfying the target fill factor (FF) within a scanning range that exceeds 80% of the scanning amplitude of the maximum amplitude. MATLAB analysis was used to facilitate frequency selection for the HDHF Lissajous scanning. Algorithm for the MATLAB analysis based on the following equation, $N \geq N_{MIN}$ (FF), where FF is the target fill factor, N is ($f_x + f_y$) / GCD.

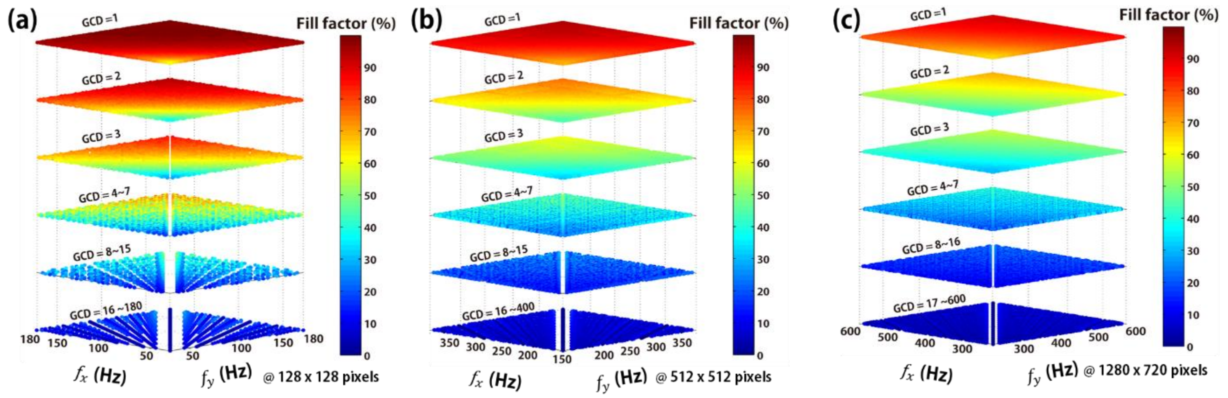

Figure S1. Scatter plots of the fill factor for 128 x 128, 512 x 512m and 1280 x 720 image pixels depending on scanning frequencies. (a)The fill-factor for a single shot image of 128 x 128 pixels depending on different scanning frequencies from 30 Hz to 180 Hz. (b) The fill factor for a single shot image of 512 x 512 pixels depending on different scanning frequencies from 150 Hz to 400 Hz. (c) The fill factor for a single shot image of 1280 x 720 pixels depending on different scanning frequencies from 200 Hz to 600 Hz.

Table S1. The minimum total lobe number (NMIN) for various image pixels depending on the fill factor

F (%)	128 x 128	256 x 256	512 x 512	1280 x 720
61	61	121	250	463
62	63	124	255	477
63	65	127	263	490
64	66	131	270	505
65	68	135	279	517
66	70	138	287	530
67	72	141	294	543
68	74	145	302	556
69	76	149	310	570
70	78	154	320	581
71	80	158	328	595
72	83	164	338	613
73	85	167	347	629
74	87	172	356	649
75	90	177	365	668
76	92	181	375	689
77	95	186	385	709
78	98	192	393	731
79	100	197	405	752
80	103	204	416	778
81	107	209	428	803
82	110	216	441	829
83	114	223	457	857
84	117	228	471	884
85	120	237	488	911
86	125	244	504	944
87	130	254	525	976
88	135	262	547	1009
89	140	271	571	1045
90	146	284	594	1082
91	152	297	621	1126
92	159	310	653	1169
93	166	323	688	1214
94	176	339	727	1273
95	186	358	769	1346
96	198	377	816	1436
97	214	401	875	1549
98	232	432	958	1785
99	260	473	1081	1943
100	269	528	1121	2116

2. Fabrication of Lissajous fiber scanner.

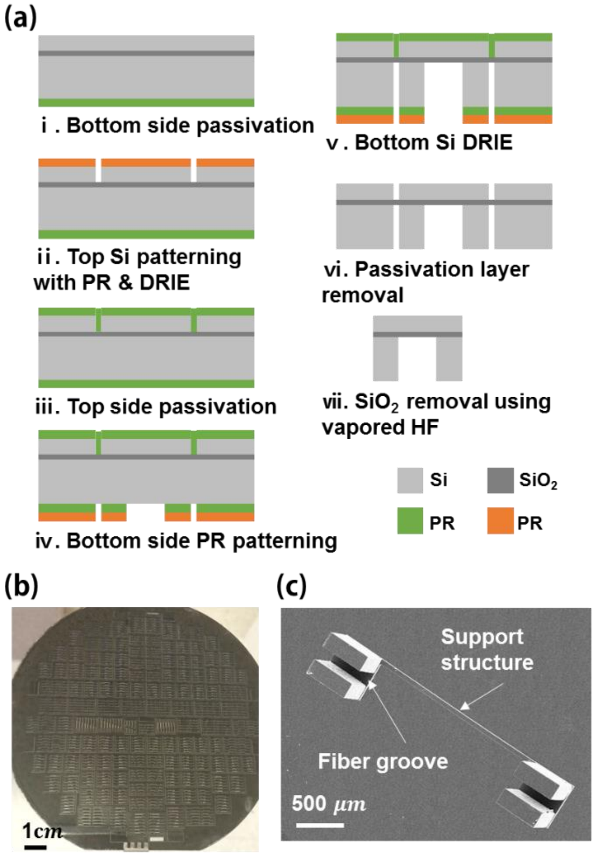

Figure S2 Fabrication of Lissajous fiber scanner. (a) Microfabrication procedure of the microtethered silicon oscillator (MTSO). First, the bottom side of 6-inch a silicon-on-insulator wafer was passivated with photoresist. Second, the MTSO were photolithographically defined with photoresist and etched by a DRIE process. The top side of the SOI wafer was then passivated with a thick photoresist. Third, a silicon grooves for microassembly with a single optical fiber and the MTSO was further etched by using DRIE on the bottom silicon layer. After oxygen plasma ashing of the passivation photoresist layer, the silicon structures were released by removing the buried oxide layer in vapored hydrogen fluoride. Individual MTSOs were finally separated from the SOI wafer by breaking off the silicon tethers with Joule heating. (b) The fabricated SOI wafer. (c) SEM image of the fabricated microspring.

3. Frequency selection for HDHF Lissajous scanning

Resonant frequencies of the scanner, which obtained Fig. 4 (c-f), are 1110 Hz and 951 Hz for each axis, respectively. The frequency ranges for bi-directional scanning frequencies of the scanner are set to the resonant frequencies of ±5 Hz, which are frequency ranges that exceed 80% of the scanning amplitude of the maximum amplitude. The fill factor and the frame rate were initially set to be over 80 % and 10 Hz for 256 x 256 pixels, respectively. Based on Table 1, the minimum total lobe number is 204 and the target frame rate of 10Hz is the same as the GCD. According to equation 1, N_{MIN} (80) = 204 ≤ (f_x + f_y) / 10. Fig. S3a displays a color-map of total lobe number depending on scanning frequencies within the frequency range, where the maximum (N ≥ 2,000) and the minimum (N ≤ 204) indicates yellow to black in color, respectively. Fig. S3b also describes a color map of the GCD depending on the scanning frequencies. Some specific areas that N is below 204 are filled in black color. The scanning frequencies for HFHD Lissajous scanning were finally set to be 1,110 Hz and 950 Hz for N=206 and GCD=10.

When the micro-scanner is driven by the sinusoidal signals including scanning frequencies, the fill factor shows a little variation depending on the phases of driving signals. The fill factor can be maximized simply by a little adjusting the phases at a function generator.

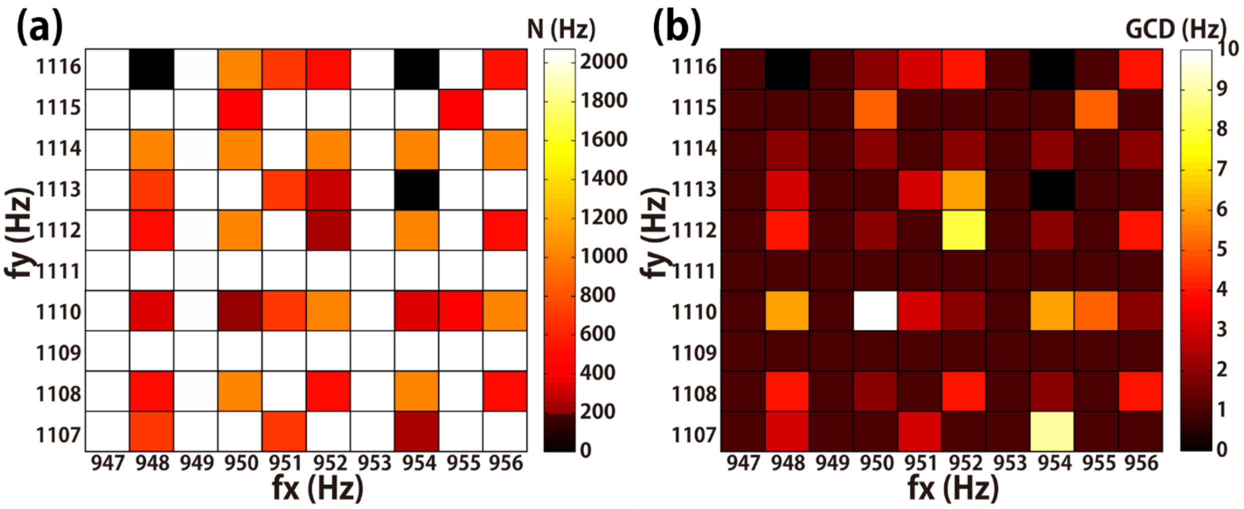

Figure S3. A color-map of total number of lobes, where the maximum (N ≥ 2,000) and the minimum (N ≤ 204) indicates yellow to black in color, respectively. (h) A color map of the GCD based on the second requirement. Some specific areas that N is below 204 are filled in black color to satisfy satisfy the above equation. The scanning frequencies for HFHD Lissajous scanning were finally set to be 1,110 Hz and 950 Hz for N=206 and GCD=10.

4. Confocal imaging system setup

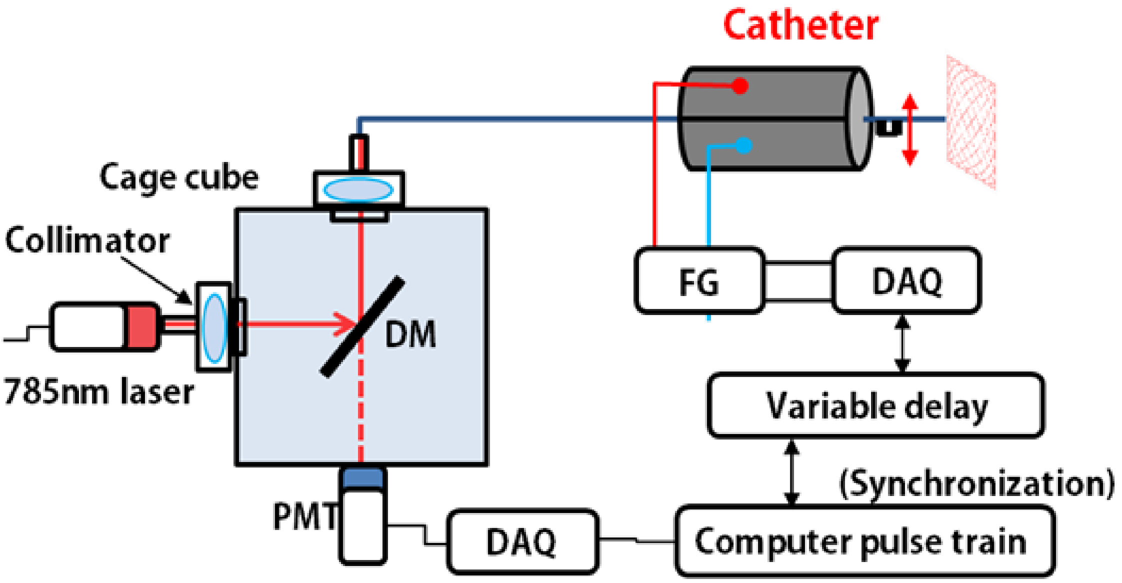

Figure S4. A schematic of confocal imaging system setup.

5. HDHF Lissajous scanning for 20 frame rate and 40 frame rate by simulation.

The frequency selection rule for HDHF Lissajous scanning is a general rule to satisfy high definition and high frame-rate within limited scanning frequencies range. Consequently, the selection rule can offer not only 10 frame-rate, but also over 10 frame rate. For example, 20 frame-rate and 40 frame-rate can be satisfied by using ~ 2 kHz and ~5 kHz scanning frequencies with over 96 % at 256 x 256 image pixels, respectively. Figure S4 shows scanning patterns satisfy 20 frame rate and 40 frame rate depends on measuring time by simulation.

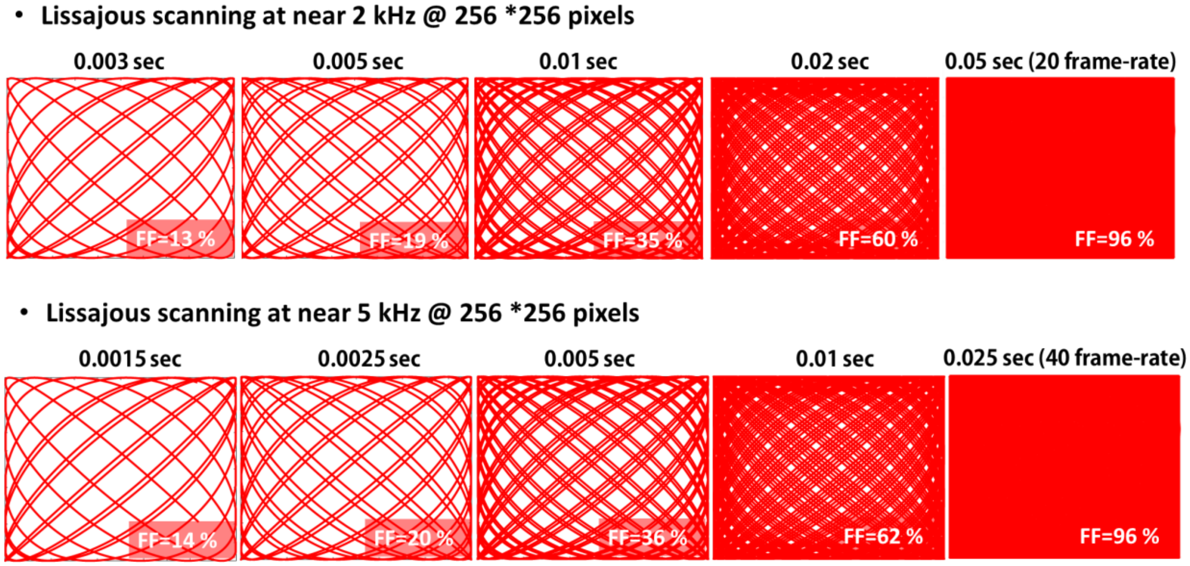

Figure S5. Scanning patterns satisfy 20 frame rate and 40 frame rate depends on measuring time by simulation.
